# Conservative Treatment of Postinfarction Left Ventricular Free Wall Rupture

**DOI:** 10.1155/2020/8832578

**Published:** 2020-09-17

**Authors:** Anh Tuan Vo, Thu Trang Nguyen, Thanh Thuy Tran, Dinh Hoang Nguyen

**Affiliations:** Department of Cardiovascular Surgery, University Medical Center, University of Medicine and Pharmacy at Ho Chi Minh City, Ho Chi Minh City, Vietnam

## Abstract

Left ventricular free wall rupture is a fatal complication of acute myocardial infarction. Emergency surgical repair is usually indicated to treat this condition. However, in very high surgical risk cohort, conservative treatment can also be considered. We report a case of left ventricle pseudoaneurysm that was successfully treated conservatively in a 4-year period.

## 1. Introduction

Postinfarction rupture of the left ventricular free wall is a rare complication but often fatal. Emergent surgical repair is usually the treatment of choice. However, in very high-risk patient with stable hemodynamics, conservative therapy might also be an alternative therapy. We report a case of left ventricular free wall rupture that survived with medical treatment alone.

## 2. Case Report

An 87-year-old man was admitted to the emergency department of a local hospital due to orthopnea and severe retrosternal chest pain radiating to his jaw. The patient had a past history of myocardial infarction (10 years ago), paroxysmal atrial fibrillation, hypertension, type 2 diabetes mellitus, and stage D chronic obstructive pulmonary disease (COPD). After three days of medical treatment, the patient's condition did not improve; he was then transferred to our hospital.

On admission, the patient was conscious with a heart rate of 125 bpm, irregular rhythm, and blood pressure of 140/80 mmHg, and room air oxygen saturation was 93–95%. On physical examination, auscultation revealed irregular and distant heart sounds, radial and femoral pulses were normally detected, and breath sounds were good bilaterally. A 12-lead ECG showed an atrial fibrillation with moderate ventricular response, QS wave in V1–V4, and no ST elevation detected. Initial cardiac enzyme was found to be nearly normal with Troponin T level of 0.016 ng/mL and CKMB level of 28 U/L (normal range: <25 U/L).

**Figure 1:**
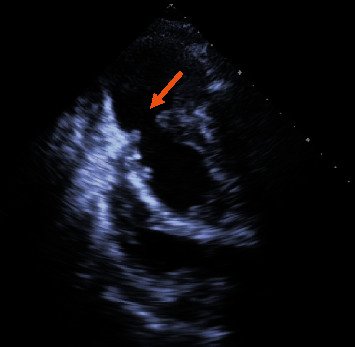
Left ventricular pseudoaneurysm formation, the perforation located at the apex with the size of 19 mm.

Emergency transthoracic echocardiography (TTE) showed a massive pericardial effusion with maximum diameter of 38 mm without tamponade signs, mild mitral regurgitation, and severe left ventricular dysfunction with an ejection fraction of 27%. The anterolateral wall of the left ventricle near the apex was ruptured and the left ventricular chamber connected to the pericardium sac through a hole of 19 mm in diameter. The perforation was covered by a thrombus and thus created a false aneurysm with a diameter of 40 mm × 30 mm (Figure 1).

The patient and his family were informed about the critical situation. Due to the very high surgical risk, the family refused surgical treatment and accepted the risk of sudden death. The patient was discharged 3 days later, when his blood pressure and heart rate were stabilized (on July 2016). The medical treatment on discharge included low dose beta blocker and angiotensin-converting enzyme inhibitor (ACEi) to keep the systolic blood pressure around 100 mmHg. No anticoagulation/antiplatelet therapy was prescribed at this moment, and a prolonged bed rest was recommended.

**Figure 2:**
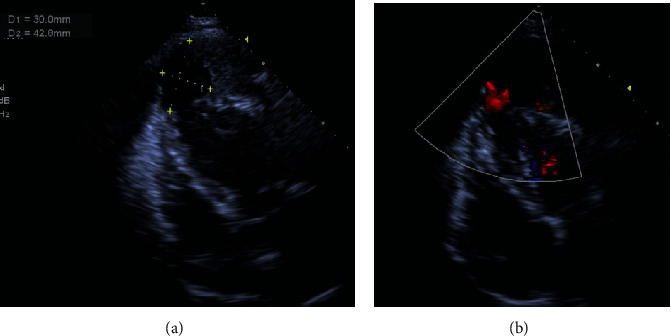
Four-chamber view TTE: the pseudoaneurysm did not change in the size (a) and active bloodstream to the pseudoaneurysm on color Doppler (b).

The patient was then followed up by a local cardiologist. Warfarin was initiated 6 months later, and the International normalized ratio (INR) was maintained around 2.0. After 45 months, the patient still survives the disease. He can now perform personal activities by himself. The last echocardiography performed on March 2020 showed that the pericardial effusion size decreased dramatically, and the old pseudoaneurysm was reinforced by a large thrombus in the pericardial sac. Color Doppler showed an active blood flow between the pseudoaneurysm and left ventricle (Figure 2).

## 3. Discussion

Postinfarction rupture of the left ventricular free wall is a catastrophic complication. It consists of about 4.7% of myocardial infarction cases according to a report [1]. The incidence of ventricular rupture after myocardial infarction has reduced when percutaneous coronary intervention revascularization developed. The risk factors for postinfarction left ventricular free wall rupture include age greater than 60 years, female gender, preexisting hypertension, absence of left ventricular hypertrophy, first myocardial infarction, and midventricular or lateral wall transmural infarctions [2].

Rupture of anterolateral wall is not commonly seen. A case report of 290 patients with left ventricular pseudoaneurysms showed that the rupture located most commonly at posterior site (43%), followed by lateral (28%), apical (24%), inferior (19%), anterior (18%), and basal area (14%) [3].

Left ventricular rupture is the third cause of death in acute myocardial infarction, accounted for 4-24% among necropsy cases [4]. Nowadays, the surgical treatment is preferred to conservative treatment, but the postoperative mortality and morbidity remain high [5]. Medical treatment and less invasive modalities, such as percutaneous fibrin-glue injection, have also been reported, but the outcomes are still controversial [6]. Our patient survived the primary phase of left ventricular rupture owing to the subacute formation of a pseudoaneurysm rather than a blow-out rupture, which prevented the leakage of blood to the pericardial cavity. And medical treatment was decided based on lack of hemodynamic instability, advanced age, accompanying comorbidities, and substantially high surgical mortality. Conservative treatment included prolong bed rest, beta blockers, and strict blood pressure control to minimize the pseudoaneurysm rupture risk. However, the prognosis of this pseudoaneurysm is unpredictable, and sudden death can occur [6]. Nevertheless, he has no significant health issues after 4 years follow-up. Long-term survival was reported by Moreno et al., in a series of 10 patients, with a survival rate at 4 years as high as 74.1% [7]. On the other hand, the outcome of patients with this condition treated conservatively has been assumed to be poor, with a 2-year mortality of around 50% in one series [8].

In conclusion, postinfarction left ventricular rupture is a usually fatal condition, and surgery is the treatment of choice with a fairly high morbidity and mortality. In very high-risk patient, conservative treatment could be considered, but the long-term outcome is unpredictable.
